# The role of the sports coach in developing athlete capacity for transition to retirement

**DOI:** 10.3389/fspor.2025.1650578

**Published:** 2025-08-15

**Authors:** Deborah Agnew, Shane Pill

**Affiliations:** College of Education, Psychology and Social Work, Flinders University, Adelaide, SA, Australia

**Keywords:** coach, athlete retirement, democracy in action, athlete centred coaching, 3+1Cs

## Abstract

The transition from high performance athlete to retirement can present significant challenges for athletes and requires support to navigate the process successfully. Given the central role of the coach in athletes' lives, we are suggesting expanding the perspective on athlete-centre coaching to include life and career transitions thereby extending its temporal scope beyond competitive performance. While the holistic development of the athlete is emphasised, much of the literature on athlete-centred coaching focuses on preparing the athlete through a player-centred approach for competition rather than the role of the coach in athlete transition to retirement. Therefore, this perspective piece expands the notion of coaching life skills in athlete-centred coaching by Griffen and colleagues to include the important role of the coach in developing athlete capacity for transition to retirement.

## Introduction

Transitions in sport are inevitable (1) and can occur into, through or out of sport. Transition to retirement from professional or elite sport has received significant attention in the extant literature (c.f. 2–8). It is considered a major life transition (9). The negative effects of transition to retirement as well as retirement after elite sport are well documented and include identity loss, a change in social networks, risks to both physical and mental health and a lack of subsequent career desire (10). Athletes may also experience grief, impaired sleep, body dissatisfaction, eating disorders and substance misuse (10). One of the challenging aspects that can be a barrier to preparing for life after sport is that being an elite athlete can lead to strong identification with the athletic identity and an all-consuming lifestyle with the demands of sport overshadowing other aspects of the athlete's life (11). Strong identification with the athletic identity and lifestyle can lead the athlete to underpreparing for life after sport. Around 20% of retiring athletes experience a “crisis transition” (7, 9), with between 18%–39% of athletes experiencing increased depression and anxiety symptoms (12–14). Zimet and colleagues (15) suggest these figures could be even higher as their research found up to 60% of retiring athletes report significant concerns with their mental health. We acknowledge, that despite these statistics, retirement from sport can also lead to a time of self-discovery, relief from the pressures of elite sport, and stable or improved quality of life (10). What this shows is that not every athlete's experience of transition to retirement is the same.

Most of the retirement from sport literature concentrates on the experiences of the athlete, support programs available and coping mechanisms of the athlete (6, 16). An underexplored area in the retirement transition literature is the role of significant others such as parents, partners and coaches in the retirement transition process. The role of the coach in particular is an under-researched area (17, 18). The coach is often cited as being part of the athlete's social support network, with the social support network being among the primary coping mechanisms for retiring athletes (17). Coaches have been argued to be an important part of the athlete's support networks while they are an athlete (19). Maintaining a connection with coaching staff following their retirement from sport appears to be a facilitator for a smoother transition out of sport for retiring athletes (10).

It is evident that the coach could have an important role in the transition out of sport experience for athletes, yet previous research suggests that coaches may avoid discussion about retirement with the athlete (20) as they perceive the responsibility for planning for retirement as sitting with the athlete (18). However, Clowes and colleagues (19) assert that coaches have a responsibility to make sure athletes are aware of programs that promote life balance because a more balanced approach may assist in alleviating potential crisis transitions if the athlete is underprepared for retirement. It is acknowledged that pre-retirement planning and education can facilitate a smoother transition out of sport (16, 21, 22). While we propose an athlete-centred coaching philosophy explained through the model of Griffin and colleagues (23) can frame the important role of coaches in helping athletes prepare for and transition to retirement we recognise that coaches have been found to actively discourage athletes from pursuing interests outside of sport (17). For example, coaches may not encourage pursuits such as additional career opportunities or education, if they believe that the outside pursuits will impact the athlete's performance in the sport (17). Furthermore, the barriers imposed on athletes by coaches not to prepare for retirement and focus on current performance needs may lead to athletes excluding the coach from the decision-making process altogether (17). We suggest a more humanistic philosophy to coaching that allows for education and mentoring can enhance athlete transition out of sport (24).

While we are suggesting that coaches have an important role in assisting the athletes to prepare for life after sport, we recognise that barriers to athlete centred coaching include organisational expectations on results and coach education. In addition, the habituated experiences of a performance-oriented coach may be a barrier to the practical implementation of athlete centred coaching (25). We are mindful of Hadfield's (25) call for patience when working with changing coach's perspective about the nature of their role working with athletes. We acknowledge that the application of an athlete-centred coaching approach is not straightforward, particularly in sports that do not have continuity with their athletes such as national training programs where players may be drawn from clubs into camps and preparation and participation for specific tournaments (26).

### Athlete centred coaching

An athlete-centred coaching philosophy foregrounds a holistic perspective of the athlete as learner through athlete ownership of their outcomes with assistance from their coach. The athlete is therefore accountable for their outcomes as a player and as a person. An athlete-centred coaching philosophy is thus one of empowerment (27). It is also known as a humanistic approach addressing an athlete's physical, social-emotional, cognitive and cultural learning needs (28, 29). An athlete-centred coaching approach thus prioritises the long-term development and wellbeing of the person and the immediacy of the need to develop the person as a player (30).

**Figure 1:**
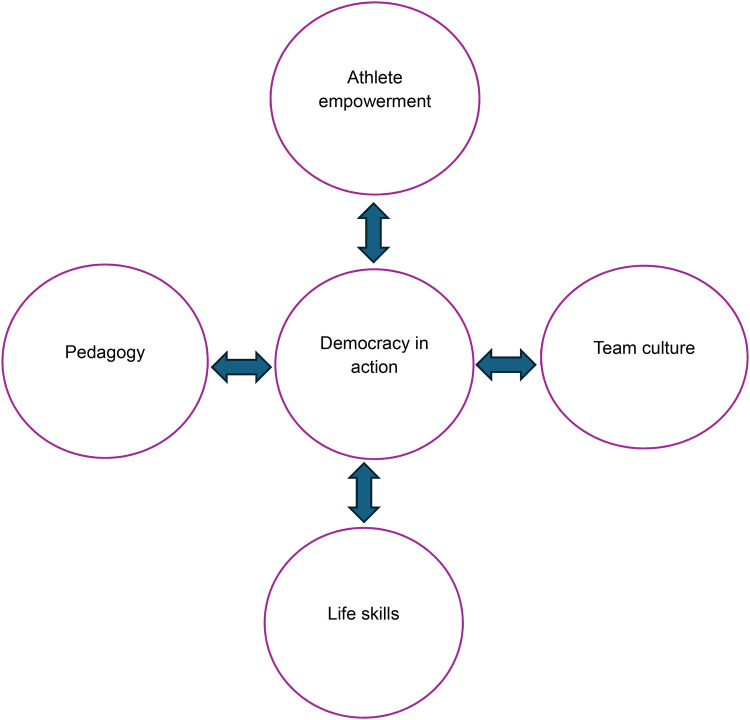
Athlete centred coaching as a holistic process (23).

Griffin and colleagues (23) (Figure 1) explain athlete-centred coaching that connects the pedagogy-coaching style, culture and climate of the sport as a holistic development of athletes called *democracy in action*. They emphasise the affective experience of coaching as most of the athlete-centred coaching literature focuses on development of athletes as thinking players through a game-based coaching approach. They also foreground athlete agency in directly influencing and owning their own learning and knowledge generation.

We suggest a modification of Griffin and colleagues (23) pedagogical principles to indicate the coach as educator in athlete-centred coaching as democracy in action:

1. Coaching as facilitating. This involves the coach setting problems with boundaries that guide exploration and the search for solutions (pedagogy);
2. Tactical complexity. This involves the use of a game-based approach for education of the tactical, technical and strategic aspects of game-sport performance (pedagogy);
3. Ongoing assessment of player learning. This involves continuous feedback enabling the athlete to reflect on and act upon to self-manage their own learning (athlete-empowerment);
4. Setting the sport culture. This involves the coach's expression of the values, norms, rituals, traditions of the team (team culture); and
5. Integrating life skills education. This involves the development of the skills that will support the athlete to thrive throughout their life (life skills).

Specific to number 5, Griffin and colleagues' athlete-centred model includes a focus on integrating the following concepts into coaching: (a) critical thinking and problem solving; (b) communication and collaboration; (c) flexibility and adaptability; (d) initiative and self-direction; and (e) social and cross-cultural skills. Not stated in Griffin and colleagues work but what we see as inherent in making their model work in practice is athlete self-regulation. Self-regulation is evident when an athlete is self-observant and self-motivated to guide their actions to achieve their goals based on self-directed change (31). Self-observation involves two functions towards self-regulation: it provides the athlete information required for realistic goal setting, and the information needed to realistically evaluate progress towards the goals (31). To assist coaches in being athlete-centred, Nilsen and colleagues (32) suggest coaches need to be taught a metacognitive perspective so that coaches can be aware of their thinking and how that thinking influences their coaching behaviour.

## Discussion

The end of an athlete's career can come from a single moment such as a career ending injury or unanticipated deselection from a team, however often the transition out of sport is part of a much longer process (11, 33, 34). Research by author 1 (35) highlights that ongoing coach support with a focus on athlete wellbeing is an important dimension of athlete-centred coaching. Athletes in this study were transitioning from elite level national sport to semi-professional state league sport. They worried about the absence of qualifications and workforce experience limiting their opportunity to find meaningful work needed as now they are part time athletes. What the athletes reflected on was the absence of continuous professional learning and guidance towards working life after professional sport. However, research finds that coaches are often reluctant to provide this guidance on life after professional sport while the athlete is still competing as they perceive the discussion about life after professional sport to be distracting to athlete performance as well as being outside of the time they have available to coach (18, 20). While leaving sports careers are disruptive to the individual (36), we found, no evidence in the literature that preparing for life after sport during an athlete's career will affect athlete performance. In fact, Lavallee (5) stated that pre-retirement planning is positively associated with aspects of performance such as team selection, length of service in a team and length of career.

Evidence from dual career research shows that athletes are better equipped for transition from sport if they attend a dual career sports program (37). The players in author 1's study (35) explained that in their elite sport environment clubs do “everything for them.” Players believed that staff, including coaches, at both the elite and the semi-professional state league level should be providing ongoing assistance with workforce preparation and in finding subsequent employment as they transition from sport. One way coaches can demonstrate an athlete-centred coaching approach is to be supportive of athletes having a working life outside of sport. Under working life, we include the undertaking of study that is preparing the athlete for life after sport.

The athlete-centred coach will play a proactive role in preparing athletes for transition from sport as they recognise and are empathetic to the fact that transition can be immensely challenging (7, 9). As an athlete-centred coach, the coach may use their relationship with the athlete to foster with them a broad personal identity which encompasses their athletic identify while extending to the development of interests, skills, and aspirations outside of sport. In this way, the athlete-centred coach prevents the athlete from developing an identity that is tied solely to their sporting career. The athlete-centred coach will be able to identify the athlete's ability to take ownership of their personal development (38) and then work from that point to develop athlete self-responsibility and therefore ongoing self-management towards what their life after sport will look like.

We are therefore arguing that the athlete-centred coach plays an active role in encouraging athletes to have a parallel path, with one path being their ongoing development as an athlete and the other path being their continuing life-skills development through exploration of potential career paths by undertaking part-time work, work experience, or further education (39). Coaches play an important role in normalising athlete preparation for transition to retirement, including framing successful transitions as a process that begins during the athlete's playing career (18). We argue that coaches claiming to be athlete-centred have an ethical responsibility to support the development of parallel paths to assist athletes to see transition from sport as a continuing process and opportunity while playing. In this way, athlete-centred coaching can be understood as democracy in action because the coach becomes a facilitator of player ownership and self-management of their learning for life after sport so that when the moment arrives the athlete understands that they have ownership of what that experience will be. We are therefore suggesting that the athlete-centred coach plays an important role through their relationship with the athlete to establish athlete ownership and responsibility for their learning and development towards the life that they want after sport.

The coach-athlete relationship is an important part of the athlete's support network (17, 19). An athlete-centred coach builds this relationship on conversations about athletic performance and the broader perspective of developing as a person. By prioritising conversations about “life”, the coach becomes a guide, mentor, and facilitator of the athlete ability to be self-determined about transition from sport as a continuing process while playing.

**Figure 2:**
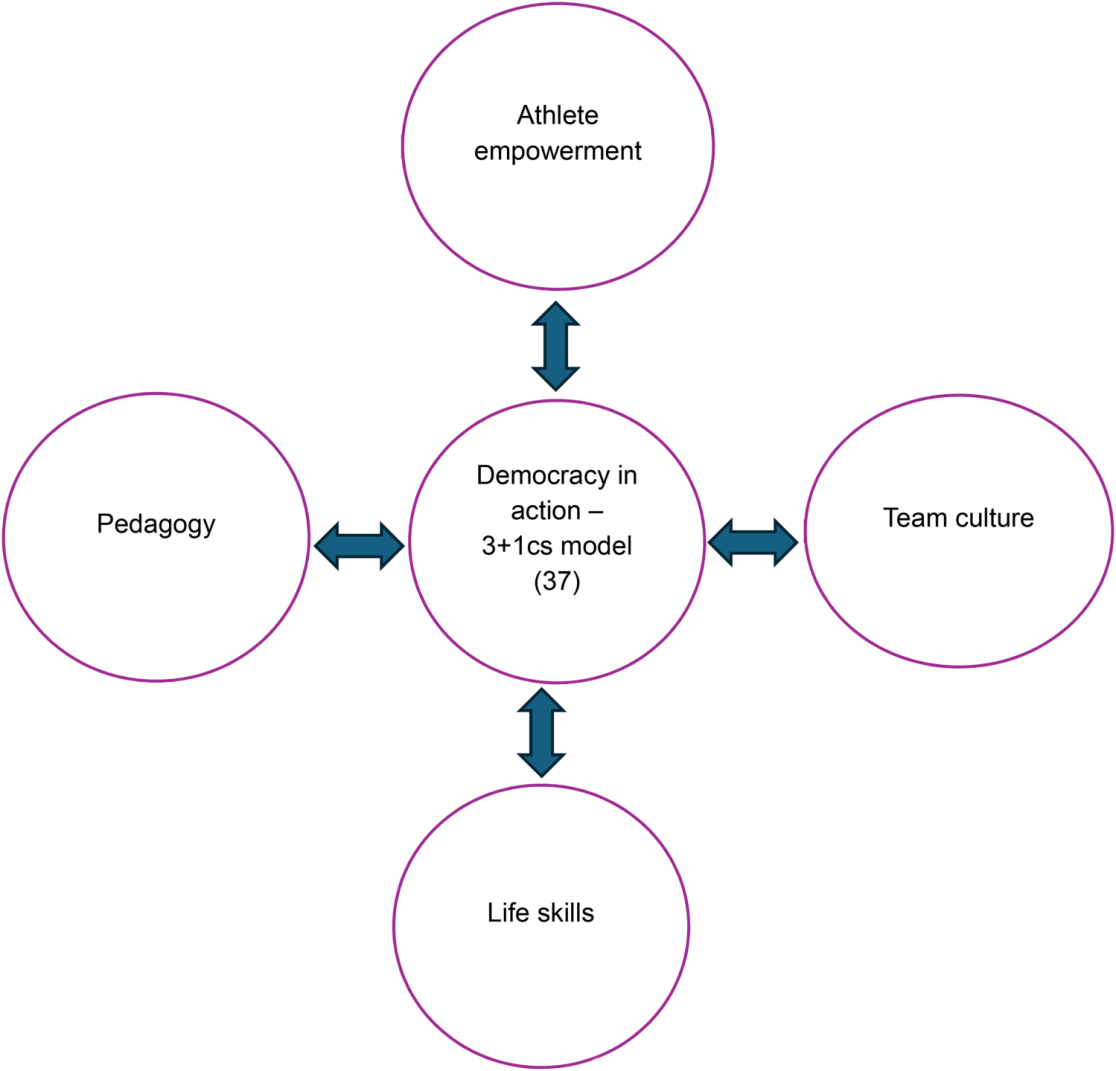
Athlete centred coaching as a holistic process incorporating the 3Cs + 1 relationship model (23, 40).

To become democracy in action regarding transition into life after sport we are suggesting Griffen et al.'s model needs an addition to frame the role of the coach-athlete relationship. We believe that Jowett's model (40) of 3 + 1Cs meets this purpose: closeness (coach emotional bond with the athlete), commitment (coach staying connected to the athlete), complementarity (coach working in cooperation and collaboration with the athlete) and co-orientation (coach developing common ground with the athlete) enables democracy in action in athlete-centred coaching to be well understood and operationalised (Figure 2).

When applied to the retirement transition process, the 3Cs + 1 model (40) offers a compelling framework for how athlete-centred coaches can support athletes who are leaving elite sport. One of the prominent strategies athletes use to cope with leaving elite sport is maintaining a connection with the sport (41, 42). Given the role of the coach as a member of the athlete's support network, having a coach who maintains communication or an emotional bond with the athlete can be a crucial part of creating throughout the playing career a supportive environment for the time transition to retirement occurs. An athlete-centred coach who maintains the type of caring relationship we are espousing with athletes can reduce the feeling that the athlete is alone in the transition out of sport. Previous research by the authors (43) posits that caring for the athletes is not purely related to wellbeing, rather is part of the creation of a caring culture in sports clubs which assists in the development of athletes as people. Coaches in this research perceived it to be their responsibility to facilitate the development of the athletes as people and not just focus on sporting skills. An important part of being an athlete-centred coach is being approachable to talk with athletes about their personal and professional-sport related concerns. Through open communication coaches may be able to identify athletes who are experiencing challenges (43), including in their preparation for life after sport.

Commitment refers to the connection between the athlete and coach which is maintained over time. A key component of the athlete-centred coaching approach is having a holistic approach to coaching which recognises the whole person and not just the athlete (23). Coaches can demonstrate a commitment to the athlete through acknowledging their outside interests and pursuits and enabling a training ecosystem that incorporates the development of life skills that are transferrable beyond sport.

An athlete-centred coach who can adapt to incorporating a holistic approach can enable the athlete to build confidence in outside pursuits. Simple adaptations that are within the coach's control include the timing of training. For example, having the same rostered day off each week, regardless of the day of competition enables the athlete to pursue a non-sporting role, or enrol in an educational pursuit (44) and achieve a more balanced lifestyle while they are playing which can contribute to a smoother transition out of sport when that moment arrives. In this way, coaches are working complementarily with the athletes to prepare for retirement. We acknowledge that such decisions may not always lie in the coach's control. There may be industrial constraints associated with workplace agreements that provide a barrier to what coaches are able to do regarding players’ rostered time off.

Finally, co-orientation involves a shared understanding between the athlete and the coach. Acknowledging that athletic careers do not last forever, and that the athlete will have a substantial portion of working life in which subsequent employment may be necessary, can empower the athlete to get prepared. Conversely, if coaches avoid discussions about life skill development which will enable flourishing in life once retired, athletes may not have the support and direction they need to pursue life skills development.

Utilising the 3Cs + 1 model (40) as a guide, the role of the athlete-centred coach is to provide stability and a sense of security through a strong athlete-coach relationship. Having a shared understanding of the needs of the athlete in both training and sport performance and a balanced lifestyle can assist the athlete to prepare over time for the challenges associated with leaving professional sport by taking self-responsibility for the management of being as prepared as possible for the process.

## Conclusion

Retirement from sport is perceived as a major life transition and can cause significant distress for athletes. In understanding the role of the coach in athlete retirement from sport through an athlete-centred approach, it is clear that coaches are a key relational figure whose role extends beyond the tactical, technical and performance driven components of coaching. We have argued in this paper that the athlete-centred coach has a central role in enabling athletes to have a parallel path which focuses on both athletic training and outside sport pursuits. Normalising preparation for life after sport represents an important aspect of democracy in action previously absent in athlete-centred coaching literature where the coach being the facilitator for athlete self-management is discussed.

We suggest that with regard to retirement from elite sport, for democracy in action to occur, Griffen et al.'s athlete-centred coaching model needs an additional component which highlights the role of the coach-athlete relationship. Adding Jowett's model (40) of 3 + 1Cs: closeness, commitment, complementarity and co-orientation facilitates democracy in action through enabling a balanced lifestyle that acknowledges the end of one's sporting career as inevitable and thus requires continuous preparation.

Coaches have an influential role in holistic athlete development; however, more work is needed to understand appropriate athlete-centred coaching as a positive influence on athlete long term development towards and into retirement. Focusing future research on athlete-centred coaching and the 3Cs + 1 model provides opportunities for advancing theory and coaching practice. For example, investigating the role of the coach in career planning and whether it enables a smooth transition out of elite sport would reflect the commitment component of the 3Cs + 1 model. In addition, the coach-athlete relationship is likely to evolve (or become disestablished) during the retirement from sport process, therefore there is an opportunity to further understand how coaches adapt their role during this important phase of an athlete's career. This article has provided a framework for using an athlete-centred approach to retirement transition. The next step is to test the framework through longitudinal studies on coach-athlete communication during transitions, or intervention studies testing training programs for coaches.

## Data Availability

The original contributions presented in the study are included in the article/Supplementary Material, further inquiries can be directed to the corresponding author.
